# Genotoxic Damage and microRNA Dysregulation in Firefighters: An Integrated Biomonitoring Case Study

**DOI:** 10.3390/jox16030078

**Published:** 2026-05-05

**Authors:** Claudia Cipollone, Riccardo Mastrantonio, Paola Mozzoni, Giada Mastrangeli, Massimo Corradi, Stefano Renzetti, Veronica Saponara, Maria Nicastro, Delia Cavallo, Raffaele Maiello, Marco Gentile, Diana Poli, Mario Muselli, Alessia Romantini, Giorgia Di Gennaro, Gloria Cenci, Carmela Protano, Matteo Vitali, Giuseppe De Palma, Cinzia Lucia Ursini, Leila Fabiani

**Affiliations:** 1 Occupational Medicine Unit, Abruzzo Local Health Authority No. 1, 67100 L’Aquila, Italy; ccipollone@asl1abruzzo.it; 2 Department of Life, Health and Environmental Sciences, University of L’Aquila, 67100 L’Aquila, Italy; giada.mastrangeli@graduate.univaq.it (G.M.); mario.muselli@univaq.it (M.M.); alessia.romantini@graduate.univaq.it (A.R.); leila.fabiani@univaq.it (L.F.); 3 Department of Medicine and Surgery, University of Parma, 43126 Parma, Italy; paola.mozzoni@unipr.it (P.M.); massimo.corradi@unipr.it (M.C.); stefano.renzetti@unipr.it (S.R.); veronica.saponara@studenti.unipr.it (V.S.); maria.nicastro@unipr.it (M.N.); 4 CERT—Center of Excellent Research in Toxicology, University of Parma, 43126 Parma, Italy; 5 Department of Occupational and Environmental Medicine, Epidemiology and Hygiene, Italian Workers’ Compensation Authority—INAIL, Monte Porzio Catone, 00078 Rome, Italy; d.cavallo@inail.it (D.C.); r.maiello@inail.it (R.M.); ma.gentile@inail.it (M.G.); d.poli@inail.it (D.P.); giorgia.digennaro@uniroma1.it (G.D.G.); c.ursini@inail.it (C.L.U.); 6 Department of Public Health and Infectious Diseases, Sapienza University of Rome, 00185 Rome, Italy; carmela.protano@uniroma1.it (C.P.); matteo.vitali@uniroma1.it (M.V.); 7 Department of Chemistry, Life Sciences and Environmental Sustainability, University of Parma, 43124 Parma, Italy; gloria.cenci@unipr.it; 8 Consiglio Nazionale delle Ricerche—Istituto dei Materiali per l’Elettronica ed il Magnetismo (CNR-IMEM), 43124 Parma, Italy; 9 Unit of Occupational Health and Industrial Hygiene, Department of Medical and Surgical Specialties, Radiological Sciences and Public Health, University of Brescia, 25123 Brescia, Italy

**Keywords:** DNA damage, firefighting, firefighter, human biomonitoring, oxidative damage, miRNA, epigenetic effect, occupational exposure

## Abstract

Firefighters are potentially exposed to multiple harmful substances, and their activities are classified as carcinogenic to humans. This case study assessed early genotoxic damage (fpg-comet and BMCyt assays) and epigenetic alterations (seven circulating miRNAs) in 35 firefighters compared to 45 non-exposed workers. Occupational exposure to fire smoke was self-reported via questionnaire. Firefighters showed higher median genotoxic DNA damage with respect to the non-exposed group (%DNA tail Buff 19.4 vs. 16.8; %DNA tail Enz 22.2 vs. 19.3; Tail moment 5.5 vs. 4.5; % of apoptotic cells 1.13 vs. 0.97). miRNAs related to air pollution, oxidative stress, tumor suppression, and immune responses, like mir-16, mir-15a, mir-29a, mir-125b, and mir-142, showed significant downregulation (*p* < 0.001) in the exposed group. Mean percentages of early apoptosis biomarkers and composite DNA damage indices among FF also differed significantly from the other participants (‰Condensed chromatin 0.46 vs. 0.06; ‰Tot anomalies 5.15 vs. 3.82). Multiple correlations emerged, particularly between miRNAs and comet assay parameters, and between comet assay and BMCyt indicators. The implemented integrated approach provides information about the existence of a relationship between genotoxic and epigenetic effects in firefighters, also influenced by time since exposure. Future studies with bigger sample sizes are required.

## 1. Introduction

Firefighters (FF) are occupationally exposed to a complex mixture of potentially genotoxic agents, including combustion products, polycyclic aromatic hydrocarbons (PAHs), formaldehyde (FA), volatile organic compounds, metals, and particulate matter. These xenobiotics are often in combination with heat stress, circadian disruption, and other physical stressors [1]. Over the past decades, epidemiological evidence has increasingly suggested an elevated risk of several cancers among FF, including malignancies of the respiratory tract, hematological neoplasms, and other solid tumors. In 2023, the International Agency for Research on Cancer (IARC) classified occupational exposure in FF as carcinogenic to humans (Group 1) [2,3]. Although cancer risk has been studied at an epidemiological level, considerably fewer investigations have focused on early biological effects and intermediate biomarkers of genotoxicity in this occupational group.

Biomarkers of early DNA and chromosomal damage can offer an opportunity to detect subclinical effects of exposure before overt disease develops and to characterize inter-individual susceptibility. Cytogenetic assays such as the micronucleus (MN) test and DNA strand-break measurements by the comet assay are widely used as indicators of genotoxic damage in occupational and environmental studies [4]. With regard to FF, recent studies applying MN in buccal and urothelial cells and comet assays in peripheral blood cells have provided interesting results, suggesting that FF’ occupational exposure may induce genomic instability [5,6,7]. Moreover, in the literature, we found limited use of minimally invasive cytogenetic endpoints such as MN in buccal cells among these types of workers. This biomarker can assess genotoxic effects at epithelial barriers that are directly exposed to inhaled contaminants [7].

Small non-coding microRNAs (miRNAs) have emerged as promising biomarkers of exposure and early effect in relation to environmental and occupational toxins. MiRNAs regulate gene expression post-transcriptionally and are involved in key pathways related to DNA damage response, oxidative stress, inflammation, and cell cycle control [8,9]. Changes in circulating miRNA profiles have been documented in populations exposed to air pollution, combustion products, and other genotoxic agents. Specific miRNA signatures have been linked to early molecular events [10]. The present case study focused on seven miRNAs (miR-16, miR-15a, miR-10b, miR-181a, miR-29a, miR-125b, and miR-142-3p) selected for their involvement in biological pathways linked to occupational exposure to combustion-derived pollutants, including inflammation, oxidative stress, immune dysregulation, and carcinogenesis [11,12]. miR-16 and miR-15a regulate cell cycle and apoptosis and are recognized biomarkers of cellular stress and environmental exposure [13], with documented alterations in response to air pollution and oxidative stress [10,14]. miR-10b and miR-181a are associated with immune regulation and inflammatory processes, and their dysregulation has been reported in conditions of chronic inflammation [15,16]. miR-29a and miR-125b are involved in epigenetic regulation, fibrosis, and tumor suppression, and have been proposed as biomarkers of susceptibility to environmentally induced diseases [17,18,19]. Finally, miR-142-3p reflects immune activation and dysregulation, with circulating levels linked to inflammatory and environmental responses [20,21].

Recent articles investigated miRNA-based biomarkers in FF [22,23,24]; despite this, their relationship with established genotoxicity endpoints such as MN frequency and DNA strand breaks remains largely unexplored [25]. Studies about genotoxic effects among FF share the same limitations: they consider only one genotoxic endpoint at a time and do not investigate it in relation to epigenetic markers [5,6,7,26].

Against this background, the aim of the present exploratory study was to assess the genotoxic and epigenetic effects caused by the firefighting activities. To achieve this, classical cytogenetic and DNA damage biomarkers were assessed together with emerging molecular endpoints. Specifically, we assessed micronuclei and other cellular anomalies in buccal epithelial cells by Buccal Micronucleus Cytome assay, DNA strand breaks, and oxidized DNA damage using the fpg-comet assay in peripheral blood cells, and the expression of selected miRNAs in blood. This integrated approach and the analysis of the relationship between the different endpoints represent the novelty of this study. In addition, we collected detailed information on individual habits and anamnestic history, including occupational variables, lifestyle factors, and medical conditions, to account for potential confounding and to explore their influence on genotoxic and epigenetic endpoints. By integrating these complementary biomarkers, our aim is to lay the groundwork for understanding early genotoxic and epigenetic effects associated with firefighting activities. We also aimed at highlighting possible associations or correlations between them and contributing to the development of more effective strategies for occupational health surveillance in this high-risk population.

## 2. Materials and Methods

### 2.1. Study Design

The present case study is part of a wider one, focused on investigating occupational exposure to FA and its effect on the health of healthcare workers, industrial workers, and FF. This cross-sectional research involved a group of FF from the Provincial Command of the Firefighters of L’Aquila (southern-central Italy); the participation rate was 63.63% (35 voluntary participants out of a total of 55 workers). In accordance with the aim of the study, we collected information about early genotoxic damage by taking samples of blood and buccal exfoliated cells and by gathering information about personal and medical history. With the objective to study the relationship with individual covariates we collected details about gender (male, female), age (continuous), job seniority (continuous), company seniority (continuous), smoke (yes, no, former), BMI (continuous), fruit and vegetables consumption (“yes” for daily or frequent consumption, “no” for rare or no consumption) and grilled and smoked food consumption (“yes” for daily or frequent consumption, “no” for rare or no consumption). Lastly, we collected information about fire extinguishing in the recent period (time elapsed since the last fire, expressed as days, a continuous variable; number of fires in the last two days, continuous; number of hours spent in the last two months in extinguishing fires, continuous). Except for this last variable, the other analyses were also carried out on a group of unexposed individuals.

This case study was conducted in accordance with the Declaration of Helsinki, and it was authorized by the CETRA ethics committee, report N. 17, dated 7 March 2024.

### 2.2. Direct/Oxidative DNA Damage—Fpg Comet Assay

At the start of the Wednesday shift, qualified medical personnel collected venous blood samples from both exposed workers and controls using sterile heparinized vacutainer (BD, Plymouth, UK), at the same time as collecting exfoliated buccal cells. Samples were kept at 4 °C, transported to the laboratory on the same day, frozen at −80 °C, and analyzed within one year. Direct and oxidative DNA damage were evaluated using the comet assay modified with formamidopyrimidine DNA glycosylase (Fpg), which specifically recognizes oxidized bases, primarily 8-oxoguanine, and converts them into detectable DNA breaks through its AP-endonuclease activity [27]. Frozen whole blood samples were thawed and then processed according to the well-established procedure described by Collins [27] with minor modifications already reported in detail by Cavallo et al. 2023 [28]. All materials and chemical reagents were from Sigma-Aldrich (currently Merck Life Science, Darmstadt, Germany). For each subject, 100 randomly selected comets (either from enzyme-treated or untreated samples) were acquired (Zen 3 Blue Edition, Zeiss, Oberkochen, Germany) and analyzed using an automated image analysis system using Software IAS, Version 10 (Delta Sistemi, Rome, Italy). The mean values of three comet parameters were calculated per subject: Tail DNA%, expressing the proportion of fragmented DNA (%DNA tail Buff); Tail length (TL), representing the smallest size of migrating DNA fragments; Tail moment (TM), obtained by multiplying TL by Tail DNA%, providing a combined measure of DNA breakage. These parameters collectively reflect the ability of a genotoxic agent to fragment DNA. Direct DNA damage was assessed using mean Tail DNA%, TL, and TM values from untreated cells. Oxidative DNA damage was quantified following Collins [29], using the parameter Tail DNA% as the most reliable indicator of total DNA breaks. Oxidative damage (Fpg-sensitive sites) was calculated as the difference between Tail DNA% in Fpg-treated cells (%DNA tail Enz) and Tail DNA% in enzyme-untreated cells (%DNA tail Buff). We therefore quantified the variable “%DNA Enz − %DNA Buff (Oxidative DNA damage)”. Subjects showing values of this difference exceeding a cut-off of 4 were classified as positive for oxidative DNA damage, consistent with Cavallo et al. [30] % of subjects with (%DNA Enz − %DNA Buff) ≥ 4). Furthermore, for each subject, we analyzed 1000 cells from the fpg-untreated sample to determine the percentage of comets (% Comets) and the percentage of apoptotic cells (% Apoptotic cells). Cells undergoing apoptosis were characterized by a notably small comet head, and most of their DNA was present in the tail.

### 2.3. Buccal Micronucleus Cytome Assay (BMCyt Assay)

Exfoliated buccal cells were collected from the right and left inner cheeks using a wet toothbrush previously immersed in phosphate-buffered solution, after the subject had rinsed the mouth with water. Sampling was performed at the beginning of the Wednesday shift. All materials and chemical reagents were from Sigma-Aldrich (currently Merck Life Science, Darmstadt, Germany). The cells were suspended in 25 mL of buffer containing 0.01 M Tris–HCl, 0.1 M EDTA, and 0.02 M NaCl (pH 7), and shipped to the laboratory performing the BMCyt assay, reaching it within 24 h. Upon arrival, cells were washed twice with the same buffer. A 50 µL aliquot of the final suspension (1.5 × 10^6^–2 × 10^6^ cells/mL) was dropped onto pre-warmed slides (37 °C). After air-drying, cells were fixed in 80% methanol for 48 h. Staining was performed with 0.005% acridine orange (Sigma), and slides were examined under a fluorescence microscope at 400× magnification (Leica, Wetzlar, Germany). For each subject, at least 2000 differentiated cells were scored independently by two expert readers according to Titenko-Holland et al. (1998) [31].

The following abnormalities were recorded: micronucleated cells (MN), cells without a nucleus, binucleated cells (indicative of cytokinesis defects or arrest), cells with nuclear buds (NB), and cells with broken eggs (BE), all markers of DNA damage; cells with condensed chromatin (early apoptosis). In addition, we also calculated some composite indices such as the enumeration of cells with more than an MN, total anomalies, NB + BE, micronuclei + NB + BEs, and, finally, subjects with MN frequencies above 1.5‰ were classified as positive. This threshold was established based on the HUMNXL project [32], which reported a spontaneous MN frequency of 0.74‰ (95% CI: 0.52–1.05) across 5424 subjects from 30 laboratories. For the acridine orange staining method used here, the reported mean MN frequency was 0.98‰ (95% CI: 0.39–1.14). Therefore, a cut-off of 1.5‰ was chosen, lying above the upper confidence limits of both estimates.

### 2.4. Blood Collection

Three milliliters of peripheral blood were collected in EDTA-containing tubes (Becton, Dickinson and Company, Franklin Lakes, NJ, USA). The aliquot intended for miRNA analysis was processed within two hours and centrifuged at 3000 rpm for 20 min. The plasma was then removed and centrifuged again at 10000 rpm for five minutes in order to remove any cellular contamination. To exclude hemolysis in plasma, the samples were thoroughly inspected visually.

### 2.5. miRNA Isolation and Quantification

RNA was extracted from 500 μL of the plasma samples using the mirVana PARIS Isolation Kit (Thermo Fisher Scientific, Inc., Waltham, MA, USA), in accordance with the manufacturer’s instructions, and quantified using a microplate reader (Varioskan LUX, Thermofisher, Inc.).

Then, the total RNA was reverse transcribed using a TaqMan™ Advanced miRNA cDNA Synthesis Kit (Thermo Fisher Scientific, Inc.), according to the manufacturer’s instructions. Briefly, the universal cDNA synthesis involved four sequential enzymatic steps: Poly(A) tailing: a poly(A) tail was added to the 3′ end of the mature miRNAs; Adapter ligation: an adapter sequence was ligated to the 5′ end of the miRNAs to provide a universal priming site; Reverse transcription: the modified miRNAs were reverse transcribed into cDNA using universal RT primers; miR-Amp amplification: to increase the quantity of the target cDNA, a universal pre-amplification step was performed using the miR-Amp Master Mix and universal primers to uniformly amplify all miRNA targets. Following the miR-Amp reaction, the cDNA was diluted 1:10 in 0.1× TE buffer. The resulting cDNA was stored at −20 °C until further use for quantitative real-time PCR (qRT-PCR).

Quantitative real-time PCR was performed using TaqMan™ Advanced miRNA Assays (Thermo Fisher Scientific) on a QuantStudio 7 Flex Real-Time PCR System (Thermo Fisher Scientific). Each PCR reaction (20 µL total volume) consisted of 5 µL of diluted cDNA, 10 µL of TaqMan™ Fast Advanced Master Mix (2×), 1 µL of the specific TaqMan™ Advanced miRNA Assay (20×), and 4 µL of nuclease-free water.

The thermal cycling conditions consisted of one step at 95 °C for 10 min, followed by 40 cycles of 95 °C for 15 s and 60 °C for 1 min. All assays were performed in duplicate, and one no-template and two internal controls were used in each experiment. The Cq values of the target miRNAs (hsa-miR-16-5p Assay ID: 477860_mir; hsa-miR-15a-5p Assay ID: 477858_mir; hsa-miR-10b-5p Assay ID: 478494_mir; hsa-miR-181a-3p Assay ID: 479405_mir; hsa-miR-29a-3p Assay ID: 478587_mir; hsa-miR-125b-5p Assay ID: 477885_mir; hsa-miR-142-3p Assay ID: 477910_mir; Thermo Fisher Scientific) were normalized to exogenous cel-miR-39 (Assay ID: 478293_mir 5′-UCACCGGGUGUAAAUCAGCUUG-3′; (Thermo Fisher Scientific).

### 2.6. Statistical Analysis

Continuous variables were summarized using mean and median as measures of central tendency, and the standard deviation together with the 1st and 3rd quartiles to describe variability. Categorical variables were reported as counts and percentages. Due to the asymmetric distribution of continuous variables, group comparisons were performed using the Wilcoxon rank-sum test for two-group comparisons and the Kruskal-Wallis test for three-group comparisons, while categorical variables were compared using the chi-squared test. The three groups considered were: Controls, Firefighters not exposed (FFNE) to fires in the previous two months, and Firefighters exposed (FFE) to fires in the previous two months. When the Kruskal-Wallis test indicated statistical significance, the Dunn test was used for post-hoc pairwise comparisons.

Multivariable regression models were applied to account for potential confounders: beta regression models were used for percentage outcomes, and gamma regression models for all other variables, given their skewed distributions. When 0s or 1s were present in the percentage outcomes, the transformation proposed by Smithson and Verkuilen [33] was applied. To evaluate the differential effect between FF and Controls on oxidative DNA damage (%DNA Enz − %DNA Buff), a beta regression mixed effects model was considered. All models were adjusted for age, sex, Body Mass Index (BMI), and smoking habits (categorized as non-smoker, ex-smoker, or current smoker).

To assess possible correlations between the three different indicators, a Spearman’s rank correlation analysis was performed. The results are shown as correlation matrices, both between the exposed and the non-exposed groups.

All statistical tests were two-sided, with a significance level of 5%, and *p*-values were adjusted for multiple comparisons using the false discovery rate method. Analyses were conducted using R (version 3.5.2).

## 3. Results

### 3.1. Study Population and Exposure Characteristics

A total of 80 subjects were included in the study, comprising 45 non-exposed workers (CTRL) and 35 FF. Compared with controls, FF were predominantly male (97.1% vs. 44.4%, *p* < 0.001), older (mean age 48.1 ± 7.3 vs. 43.3 ± 11.9 years, *p* = 0.033), had a longer job seniority both in the current role (*p* = 0.008) and within the organization (*p* = 0.013), and showed a higher BMI (*p* < 0.001). Smoking habits did not differ significantly between groups (Table 1).

Among FF, the median time between the last fire event and biological sampling was 26 days (IQR 8–46). In the two months preceding sampling, FF reported a median of 1 fire (IQR 0.5–1.5) and 3.5 h of exposure (IQR 2.0–6.0). Regular use of respiratory personal protective equipment was reported by 65.5% of FF (Table 1).

To better characterize the FF’s exposure, two groups were created based on the time elapsed since the last wildfire, so FF were further classified as FFE (fire exposure in the previous two months, n subsample = 26) and FFNE (subsample = 9) (Table 2).

### 3.2. DNA Damage Assessed by Comet Assay

FF showed significantly higher levels of primary DNA damage compared with controls. Specifically, FF exhibited increased %DNA in tail measured under alkaline conditions without enzyme treatment (%DNA tail Buff; *p* = 0.005), with enzyme treatment (%DNA tail Enz; *p* = 0.009), and higher Tail Moment values (*p* = 0.022). The percentage of apoptotic cells was also significantly higher in FF than in CTRL (*p* = 0.002). Oxidative DNA damage, defined using a predefined threshold (%DNA tail Enz − %DNA tail Buff ≥ 4), was observed more frequently in FF than in controls (42.9% vs. 24.4%), although the difference was not statistically significant (*p* = 0.081). In contrast, oxidative DNA damage expressed as the difference between enzyme-treated and buffer conditions (%DNA Enz − %DNA Buff) did not differ significantly between groups (*p* = 0.519). No significant differences were observed for tail length (*p* = 0.165) (Table 3).

After adjustment for sex, age, smoking status, fruit/vegetable consumption, grilled/smoked food consumption, and BMI, group status (FF vs. CTRL) remained a significant independent predictor of increased DNA damage. FF status was associated with higher %DNA tail Buff (estimate 1.27, 95% CI 1.11–1.44, *p* < 0.001), %DNA tail Enz (1.28, 95% CI 1.08–1.52, *p* = 0.005), Tail Moment (1.32, 95% CI 1.13–1.54, *p* < 0.001), TL (1.17, 95% CI 1.04–1.33, *p* < 0.010) and percentage of apoptotic cells (1.40, 95% CI 1.20–1.64, *p* < 0.001). No significant interaction between group status and oxidative DNA damage (representing the difference between %DNA tail Enz and %DNA tail Buff among groups) was observed. The studied dietary covariate related to fruit/vegetable consumption was shown to influence %DNA tail Buff, %DNA tail Enz, Oxidative DNA damage, and Tail Moment parameters, with a protective effect (Table 4).

**Figure 1:**
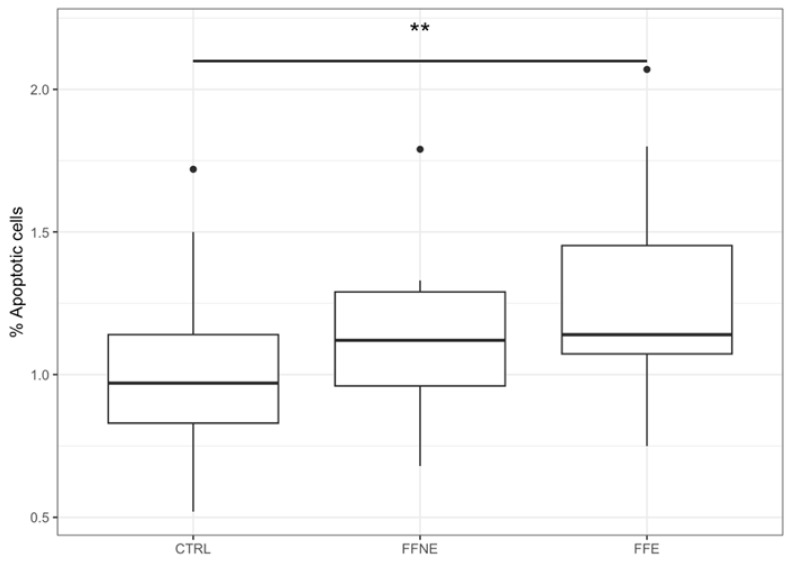
Box plot representing significant comparisons between groups (CTRL vs. FFE; CTRL vs. FFNE; FFE vs. FFNE). Notes: ** = *p* < 0.01.

When we split FF into FFNE and FFE groups (Table 5), a significant difference was observed among the three groups for % of apoptotic cells (*p* = 0.045). In particular, FFE showed significantly higher values than CTRL for the % of apoptotic cells (CTRL vs. FFE, *p* = 0.002) (Figure 1). 

### 3.3. miRNA Expression Profiles

To validate the functional relevance of this miRNA panel in the context of combustion-derived damage, we performed an in silico functional enrichment analysis using the Enrichr platform. The resulting profiles (Figures S1–S7) confirm that these miRNAs are master regulators of the cell cycle, proliferative signaling, and growth, providing a molecular link between environmental exposure and systemic dysregulation.

The molecular pathways involved in the regulation of the selected miRNAs and their interconnections are reported in the Supplementary Materials (Figures S8–S10).

**Figure 2:**
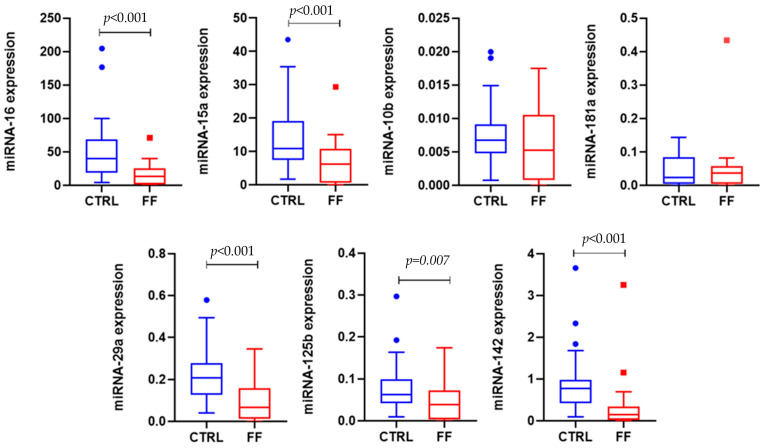
Boxplots of miRNA expression profiles in non-exposed workers and FF. Blue and red dots indicate outlayer values.

With respect to these biomarkers, the FF cohort showed a marked down-regulation of several circulating miRNAs compared with controls. Significant reductions were observed for miR-16, miR-15a, miR-142-3p, miR-29a, and miR-125b (all *p* ≤ 0.005). Specifically, the median expression of miR-16 in the FF group was markedly lower compared to non-exposed (13.941 vs. 36.575, *p* < 0.001). Furthermore, statistical analysis revealed a highly significant differential expression of miR-142 between the two study groups (*p* < 0.001): subjects in the firefighter group (FF) exhibited a marked suppression of miR-142-3p levels compared to the control group (CTRL) showing a median of 0.170 (IQR: 0.024, 0.337), whereas the CTRL group maintained a median of 0.777 (IQR: 0.415, 0.987). Similarly, miR-29a, which is frequently associated with the regulation of fibrotic processes and collagen deposition, showed almost a three-fold reduction in the FF cohort (Median: 0.070 vs. 0.207, *p* < 0.001). Finally, miR-125b expression was significantly reduced as well (*p* = 0.005), suggesting a potential molecular response to occupational exposure. No significant differences were found for miR-10b or miR-181a. After adjustment for sex, age, smoking status, and BMI, FF status remained strongly associated with lower expression levels of miR-16, miR-15a, miR-142, miR-29a, and miR-125b (all *p* ≤ 0.006). BMI was positively associated with the expression of several miRNAs (excluding miR-10b), whereas sex and smoking status showed no consistent effects (Figure 2).

As in the previous analysis, no significant differences were observed between the two firefighter subgroups based on the time elapsed since the last fire.

### 3.4. Buccal Micronucleus Cytome Assay

No significant differences were observed between CTRL and FF in MN frequency. However, FF showed significantly higher frequencies of chromatin condensation (*p* = 0.009), NB + BEs (*p* = 0,05), MN + NB + BE (*p* = 0.039), and total nuclear anomalies (*p* = 0.008) compared with controls (Table 6).

Multivariable regression analyses indicated that FF status was associated with increased chromatin condensation (OR 1.44, 95% CI 1.08–1.92, *p* = 0.014), while no significant associations were observed for micronuclei or other nuclear abnormalities.

### 3.5. Correlations Between BMcyt Assay, Comet Assay, and miRNA Expression

**Figure 3:**
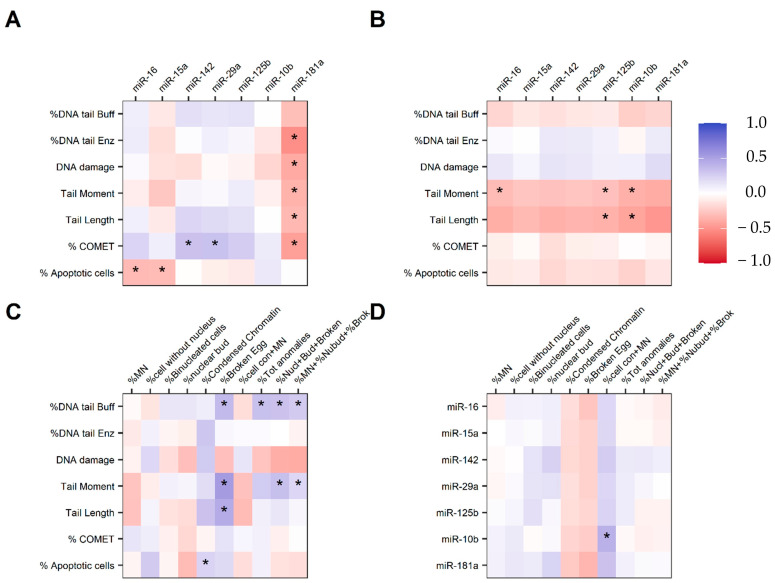
Correlation matrices showing correlations between the parameters of Comet assay and miRNAs in the CTRL (**A**) and FF (**B**) group, Comet assay and BMCyt assay in FF (**C**), and between miRNAs and BMCyt assay in the FF group (**D**). The color indicates the correlation direction and the magnitude; the star indicates statistical significance (*p* < 0.05).

Interestingly, we found several correlations between the studied biomarkers. In the control group, we only found correlations between 5 miRNAs and some parameters related to the comet assay. In contrast, in the exposed group, we found correlations involving at least one parameter for each biomarker (Figure 3A–D); we only reported analysis in which at least a statistically significant correlation was found.

Among non-exposed, with respect to the relationship between miRNAs and the comet assay parameters (Figure 3A), a negative and moderate correlation was found between miR-181a and the variables DNA tail ENZ, oxidative DNA damage, tail moment, tail length, and the percentage of comets. Apoptotic cell percentages weakly and negatively correlated with miR-16 and miR-15a regulation. Finally, the comets percentage weakly and positively correlated with miR-142 and miR-29a.

Regarding the link between miRNAs and the comet assay parameters, among FF (Figure 3B), mir-125b and mir-10b showed negative correlations, from weak to moderate, with tail moment and tail length. Tail moment is also weakly and negatively correlated with mir16.

In the FF group, we found several significant correlations between the parameters studied through the BMCyt assay and the comet assay (Figure 3C). We found positive correlations from weak to moderate between %DNA tail Buff and ‰BE and the composite indexes ‰total anomalies, ‰ NB + BE, and ‰ MN + NB + BEs. Tail moment and tail length also positively correlated with ‰BE.

Finally, in the exposed group, we found a positive and moderate correlation between mir-10b and ‰ of Cells with more than a MN (Figure 3D).

## 4. Discussion

This case study provides preliminary evidence that FF exhibit a distinct biological profile compared with unexposed workers. This profile is characterized by increased DNA damage, marked down-regulation of specific circulating miRNAs, and absence of a significant increase in stable chromosomal damage as assessed by BMCyt assay. Overall, these findings suggest that occupational exposure associated with firefighting activities elicits early molecular and cellular responses consistent with genotoxic and inflammatory stress. To the best of our knowledge, there are no directly comparable studies that applied an integrated approach using different markers. Moreover, comparable studies that could provide an interpretative framework linking genotoxic and epigenetic markers are very scarce.

Within the context of the comet assay results, FF showed significantly higher levels of primary DNA damage, as demonstrated by increased %DNA in tail and Tail Moment values under both standard alkaline conditions and enzyme-treated conditions. These findings support the hypothesis that exposure to complex mixtures of combustion-related contaminants can lead to measurable genomic damage. The significantly higher proportion of apoptotic cells observed in FF further indicates a cellular response to sustained genotoxic stress. The activation of apoptosis pathways represents a protective mechanism aimed at removing severely damaged cells, thereby preventing the stabilization of genomic alterations. The absence of significant differences in mean or median oxidative DNA damage (%DNA Enz − %DNA Buff), despite a higher proportion of FF classified as positive for oxidative damage, suggests that such lesions may be transient or partly mitigated by antioxidant defenses and DNA repair processes. Nevertheless, the persistence of increased primary DNA damage after adjustment for confounding factors (sex, age, smoking status, BMI, fruit and vegetable intake, and consumption of grilled and smoked foods) indicates that firefighter status itself may act as an independent determinant of genomic instability. A previous study [6] conducted in Portugal highlighted significant differences between FF and non-exposed participants with regard to basal DNA damage. This result appeared to be influenced by age as a confounding factor. In our sample, this characteristic was not evident. Moreover, the authors [6] identified a relevant oxidative DNA damage that was linked to the duration of exposure. We observed a clear effect on DNA among the exposed participants with respect to the non-exposed, but this association was clear both for FF that had a recent exposure to fire smoke (FFE) than for FF with no recent exposure (FFNE). Moreover, FFE also showed an increased percentage of apoptotic cells. A study from Oliveira et al. [34] reports results regarding oxidative DNA damage in this kind of worker; contrary to us, they did not find evidence of basal DNA damage. As shown in the following, our results highlighted apoptotic processes also through BMCyt assay (Table 6) than through miRNA regulation analysis (Figure 2). Diet can significantly influence overall health status. Evidence from peer-reviewed studies indicates that fruit and vegetable intake may act as a protective factor against oxidative DNA damage, with some reports showing reduced damage in comet assay outcomes [35]. However, controlled human studies have yielded inconsistent and often non-significant results, suggesting that diet alone is a relatively weak and context-dependent determinant of comet assay endpoints. In contrast, our multivariate analysis demonstrates a clear association between dietary habits, particularly fruit and vegetable consumption, and lower genotoxicity levels measured by the FPG-comet assay.

The BMCyt assay produced notable findings. Contrary to the Comet assay, no significant differences were observed between FF and controls in MN frequency, possibly indicating that the increased DNA damage did not translate into detectable stable chromosomal alterations. This result is consistent with the hypothesis that DNA repair and apoptotic mechanisms remain largely effective in preventing the fixation of chromosomal damage in the studied population. However, FF exhibited significantly higher levels of chromatin condensation and total nuclear abnormalities, suggesting the presence of early nuclear alterations associated with cellular stress and apoptosis. The independent association between firefighter status and chromatin condensation reinforces the notion that these endpoints may serve as sensitive indicators of subclinical biological effects. In contrast to our findings, Esteves et al. [7] reported a significant increase in micronuclei frequency in urothelial cells among FF, focusing on genotoxic differences related to occupational exposure to wildfire smoke. They observed that MN formation more than doubled during the wildfire season and identified significant associations between urinary hydroxylated PAHs and MN frequency in urothelial cells. A more recent study [26] supports our observations regarding cell death parameters in full-time FF, suggesting that these cytotoxic endpoints are associated with firefighting activities. However, recent smoke exposure, as assessed by self-reported questionnaires among our exposed participants, did not influence condensed chromatin levels. Notably, as previously mentioned, the comet assay revealed a higher proportion of apoptotic cells in the recently exposed group (FFE), indicating a degree of consistency between the two genotoxicity assays employed. Unlike Esteves et al. [7], our study did not include the assessment of exposure biomarkers specifically related to firefighting activities. BMCyt assay is widely used to assess genotoxic damage in chemically exposed workers [36,37], but the scientific literature addressing these cytotoxic processes in FF is limited.

Regarding epigenetic biomarkers, one of the most notable results of this case study is the downregulation of several circulating miRNAs in FF, including miR-16, miR-15a, miR-142, miR-29a, and miR-125b. These miRNAs are known to play key roles in the regulation of cell cycle progression, apoptosis, DNA damage response, and inflammatory pathways [15,16]. Their suppression may therefore reflect a molecular response to chronic low-level occupational stress. Moreover, the positive association observed between BMI and the expression of several miRNAs is consistent with the involvement of these molecules in metabolic and inflammatory processes. Overall, this association highlights the importance of accounting for anthropometric factors when interpreting miRNA profiles [38,39]. The study of Jeong et al. [14] assessed the existence of epigenetic effects associated with firefighting. The authors highlighted the up- and down-regulation of several miRNAs associated with cancers or cancer pathways. They accounted for the FF’s job seniority and compared a group of incumbent workers with a group of recruits. Furthermore, a longitudinal study published in 2021 [40] demonstrated that tumor suppressive miRNAs decreased and oncogenic miRNAs increased with exposure. Among the miRNAs identified as significantly altered in regulation, the authors found miR181a, which is one of the miRNAs analyzed in the present study. Despite our analysis, miR-181a appears not to be statistically dysregulated. Figure 2 shows a downregulation among FF with respect to non-exposed workers. Interestingly, the authors also found that some miRNA regulation was associated with employment duration and with the time elapsed since the last fire; with respect to these findings, our results didn’t show a similar trend. Recent and pertinent studies [22,23,24] confirmed that firefighting activities may dysregulate miRNA expression, both causing downregulation and upregulation. These findings are fully aligned with ours.

Restricted to the differences between genotoxic and epigenetic endpoints, the present study highlights clear variations between exposed and non-exposed individuals. This aspect has important implications for occupational risk management and the protection of workers’ health. To enhance the safety of involved FF, management should strengthen measures promoting the use of respiratory personal protective equipment and establish health surveillance protocols that include both exposure biomarkers [41] and effect biomarkers, such as those investigated in this study. Further, more structured studies with longitudinal designs are needed to better clarify the differences among the examined effect markers and to identify areas of overlap useful for an accurate interpretation of the results.

In accordance with the aim of exploring possible relationships among the three biomarkers, our correlation analysis revealed several associations. In the non-exposed group, we found a negative correlation between mir-181a, which is associated with inflammatory signaling and cellular differentiation [19], and genotoxic parameters indicating oxidative stress and DNA fragmentation. We also observed a similar trend with regard to mir-16 and mir-15a expression and % of apoptotic cells. As shown in Figure 2, irrespective of statistical significance, the regulation of these miRNAs is higher in non-exposed workers. These results can be considered predictable in a group of non-exposed workers. In contrast, we observed a weak positive correlation between the %comets indicator and the expression of miR-142 and miR-29a. The parameter that may be considered anomalous, and potentially responsible for these findings, is the high percentage of comets in this group.

As shown in Table 3, the exposed group showed higher levels of genotoxic endpoints from the comet assay with respect to the non-exposed workers. miRNA regulation is also substantially different between the groups, showing lower values among FF. The correlation analysis (Figure 3B) demonstrates that higher levels of genotoxic damage (tail moment and tail length) are associated with a downregulation of mir10b, mir 125b, and mir16. In fact, as shown in Figure 2, the exposed group showed lower median values than the non-exposed group in the expression of these three miRNAs. Scientific Literature concerning correlations between miRNAs and the genotoxic endpoints assessed through the comet assay in human populations is scarce. However, in a recent review from Mozzoni et al. [42], the authors highlighted the existence of a positive correlation between oxidative stress and miRNA expression.

With regard to possible correlations between MN frequency detected on buccal cells and genotoxic damage assessed through the comet assay on blood, we observed positive correlations between different endpoints of genotoxic damage. These findings align with the existing Literature. A study from Valles-Pardo et al. [43] showed strong and positive significant correlations between MN frequency and the comet assay parameters. Furthermore, similar findings were found in a study conducted on oral precancer and cancer patients [44]. Although we didn’t observe a correlation between MN frequency and the Comet assay parameters, we demonstrated that primary DNA damage values increase together with ‰BE and with composite indices such as “‰Total anomalies”, “‰ NB + BEs”, and “‰MN + NB + BEs” (Figure 3C) among FF. These composite indices are all indicators of genotoxic effects. In addition, the positive correlation between the percentage of apoptotic cells detected by the comet assay and the frequency of cells with condensed chromatin (an indicator of early apoptosis) indicates that apoptotic processes are induced in both biological matrices, namely, blood and buccal cells.

As shown in Figure 3D, we observed a moderate and positive correlation between the regulation of mir-10b and ‰ of cells with more than a MN. The interpretation of this finding remains unclear, since increased genotoxic damage would be expected to be associated with a downregulation of miR-10b. However, as shown in Figure 2, miR-10b expression in exposed workers does not substantially differ from that observed in the non-exposed group, and the difference is not statistically significant. Accordingly, it can be hypothesized that occupational exposure of FF does not affect the regulation of this epigenetic endpoint. Moreover, overall, the BMCyt assay revealed only a few significant differences between exposed and unexposed groups. This aspect may explain the scarcity of significant positive or negative correlations with the studied epigenetic endpoints. Contrary to our results, Feng et al. [45] highlighted a correlation between MN detected by the cytokinesis-block MN assay and epigenetic endpoints in chemical-exposed workers (vinyl chloride monomer). Despite analyzing different miRNAs with respect to us, a clear relationship between epigenetic and genotoxic effects as a consequence of occupational exposure is demonstrated. Analogously, a study involving radiation-induced epigenetic alterations [46] showed an immediate induction of MN following radiation exposure, which was paralleled by alterations in DNA methylation and miRNA expression.

In the non-exposed group, no significant correlations were observed between genotoxic endpoints measured by the comet and BMCyt assays, nor between the BMCyt assay and miRNA levels.

According to the exploratory nature of the present study, some limitations should be acknowledged. Despite several biomonitoring studies about FF reporting similar sample sizes, the relatively small number of enrolled FF may have limited statistical power for some comparisons and reduced the ability to detect subtle exposure-related effects. Second, the cross-sectional study design precludes causal inference and does not allow evaluation of temporal changes or long-term health implications of the observed biological alterations. Additionally, although major confounders were accounted for, residual confounding from unmeasured factors, such as physical activity or environmental exposures outside the workplace, cannot be excluded. Finally, the number of the analyzed miRNAs is a clear limitation. However, the convergence of findings across the different studied biomarkers (DNA damage, cytogenetic alterations, and miRNA dysregulation) strengthens the validity of the study and supports the robustness of the observed patterns. Future improvements in enrolment of FF, the assessment of a higher number of miRNAs, and a longitudinal design will make it possible to strengthen the findings of this study.

## 5. Conclusions

This study provides evidence that FF exhibit a distinct biological response profile compared with non-exposed workers, characterized by increased primary DNA damage, alterations in nuclear morphology, and consistent modulation of circulating miRNAs involved in key cellular regulatory pathways. Collectively, these findings support the presence of early molecular and cellular effects associated with occupational exposure to firefighting pollutants. With regard to both genotoxic and epigenetic endpoints, apoptotic parameters appear to be a common feature linking the results of the different assays.

The absence of a relevant number of comparable studies poses difficulties in comparing the correlations between the observed biomarkers with those reported among workers in the same sector. The joint use of genotoxicity assays and circulating microRNAs allows for a multi-layered assessment of exposure-related biological changes. It also offers a comprehensive characterization of early molecular effects in FF. This integrated strategy may enhance the detection of subclinical damage and support a deeper understanding of the mechanisms driving occupational health risks in this group. From a translational perspective, this study has direct relevance for occupational health surveillance, suggesting that such strategies may enable earlier and more sensitive identification of at-risk individuals, with potential implications for targeted prevention. At the policy level, our results support the integration of advanced molecular biomarkers into routine monitoring programs for FF, alongside strengthened preventive measures aimed at reducing exposure.

Further research involving larger cohorts, longitudinal study designs, and precise exposure characterization is needed to better define the temporal behavior of these biomarkers and to evaluate their potential prognostic value for long-term health outcomes in FF.

## Figures and Tables

**Table 1:** Study population, occupational seniority, and FF exposure characteristics. SD = Standard Deviation; Q1 = First Quartile; Q3 = Third Quartile; n.a. = not applicable.

	Total (N = 80)	CTRL (N = 45)	FF (N = 35)	*p* Value
SEX				<0.001
f	26 (32.9%)	25 (55.6%)	1 (2.9%)	
m	53 (67.1%)	20 (44.4%)	33 (97.1%)	
N-Miss	1	0	1	
AGE				0.033
Mean (SD)	45.34 (10.42)	43.31 (11.87)	48.121 (7.33)	
Median (Q1, Q3)	45 (36, 54.75)	41 (32, 54)	45 (44, 55)	
N-Miss	2	0	2	
JOB SENIORITY				0.008
Mean (SD)	10.07 (9.6)	8.48 (10.09)	12.09 (8.65)	
Median (Q1, Q3)	8 (3, 15)	3 (2, 10)	10 (6, 15.5)	
N-Miss	7	4	3	
COMPANY SENIORITY				0.013
Mean (SD)	16.25 (11.99)	13.91 (12.51)	19.57 (10.55)	
Median (Q1, Q3)	12 (5, 27)	8 (3, 25)	15.5 (10.25, 28)	
N-Miss	17	8	9	
SMOKE				0.221
N-Miss	5	3	2	
no	40 (53.3%)	20 (47.6%)	20 (60.6%)	
former	16 (21.3%)	12 (28.6%)	4 (12.1%)	
yes	19 (25.3%)	10 (23.8%)	9 (27.3%)	
BMI				<0.001
Mean (SD)	24.26 (3.56)	22.83 (3.19)	26.18 (3.13)	
Median (Q1, Q3)	24.22 (21.59, 25.88)	22.67 (20.51, 24.72)	25.59 (24.21, 28.50)	
N-Miss	5	2	3	
Fruit/vegetables consumption				0.100
No	7 (8.8%)	6 (13.3%)	1 (2.9%)	
Yes	73 (91.2%)	39 (86.7%)	34 (97.1%)	
Grilled/smoked food consumption				0.677
No	59 (73.8%)	34 (75.6%)	25 (71.4%)	
Yes	21 (26.2%)	11 (24.4%)	10 (28.6%)	
TIME ELAPSED SINCE THE LAST FIRE (DAYS)				n.a.
Mean (SD)	/	/	36.93 (44.19)	
Median (Q1, Q3)	/	/	26 (8, 46)	
N-Miss	/	/	6	
NUMBER OF FIRES IN THE LAST 2 MONTHS				n.a.
Mean (SD)	/	/	1.34 (1.43)	
Median (Q1, Q3)	/	/	1 (0.5, 1.5)	
NUMBER OF HOURS IN THE LAST 2 MONTHS				n.a.
Mean (SD)	/	/	4.34 (4.50)	
Median (Q1, Q3)	/	/	3.5 (2, 6)	
N-Miss	/	/	6	

**Table 2:** Study population and exposure characteristics. FF are grouped based on the time elapsed since the last wildfire. SD = Standard Deviation; Q1 = First Quartile; Q3 = Third Quartile; n.a. = not applicable.

	Total (N = 80)	CTRL (N = 45)	FFNE (N = 9)	FFE (N = 26)	*p* Value
SEX					<0.001
f	26 (32.9%)	25 (55.6%)	0 (0.0%)	1 (4.0%)	
m	53 (67.1%)	20 (44.4%)	9 (100.0%)	24 (96.0%)	
N-Miss	1	0	0	1	
AGE					0.092
Mean (SD)	45.34 (10.42)	43.31 (11.87)	47 (9.23)	48.48 (6.81)	
Median (Q1, Q3)	45 (36, 54.75)	41 (32, 54)	44 (41, 55.25)	46 (44, 55)	
N-Miss	2	0	1	1	
JOB SENIORITY					0.022
Mean (SD)	10.07 (9.6)	8.49 (10.1)	16.12 (12.54)	10.75 (6.75)	
Median (Q1, Q3)	8 (3, 15)	3 (2, 10)	10.5 (8.75, 23)	10 (6, 14.25)	
N-Miss	7	4	1	2	
COMPANY SENIORITY					0.043
Mean (SD)	16.25 (11.99)	13.92 (12.51)	17.83 (10.94)	20.1 (10.67)	
Median (Q1, Q3)	12 (5.5, 27.5)	8 (3, 25)	12.5 (10.25, 24.5)	17 (11.5, 29.25)	
N-Miss	17	8	3	6	
SMOKE YES/NO/EX					0.383
no	40 (53.3%)	20 (47.6%)	6 (75.0%)	14 (56.0%)	
ex	16 (21.3%)	12 (28.6%)	0 (0.0%)	4 (16.0%)	
yes	19 (25.3%)	10 (23.8%)	2 (25.0%)	7 (28.0%)	
N-Miss	5	3	1	1	
BMI					<0.001
Mean (SD)	24.26 (3.56)	22.84 (3.2)	26.58 (2.96)	26.05 (3.25)	
Median (Q1, Q3)	24.22 (21.6, 25.89)	22.68 (20.51, 24.73)	26.02 (24.12, 29.1)	25.59 (24.48, 28.15)	
N-Miss	5	2	1	2	
Fruit/vegetables consumption					0.243
No	7 (8.8%)	6 (13.3%)	0 (0.0%)	1 (3.8%)	
Yes	73 (91.2%)	39 (86.7%)	9 (100.0%)	25 (96.2%)	
Grilled/smoked food consumption					0.808
No	59 (73.8%)	34 (75.6%)	7 (77.8%)	18 (69.2%)	
Yes	21 (26.2%)	11 (24.4%)	2 (22.2%)	8 (30.8%)	

**Table 3:** DNA damage assessed by Comet assay. Differences between non-exposed and exposed workers. SD = Standard Deviation; Q1 = First Quartile; Q3 = Third Quartile.

	Total (N = 80)	CTRL (N = 45)	FF (N = 35)	*p* Value
%DNA tail Buff				0.021
Mean (SD)	18.53 (3.55)	17.55 (3.35)	19.78 (3.43)	
Median (Q1, Q3)	18 (16.17, 20.35)	16.8 (15, 20)	19.4 (18, 21.25)	
%DNA tail Enz				0.025
Mean (SD)	21.71 (5.14)	20.54 (4.81)	23.22 (5.22)	
Median (Q1, Q3)	21.4 (17.97, 25)	19.3 (17.5, 23)	22.2 (20.44, 26.85)	
Oxidative DNA damage				0.519
Mean (SD)	3.18 (4.05)	2.99 (3.41)	3.43 (4.8)	
Median (Q1, Q3)	2.8 (0.7, 4.42)	2.7 (1.1, 4)	3.4 (0.45, 5.65)	
Tail Moment				0.045
Mean (SD)	5.35 (1.4)	5.1 (1.47)	5.68 (1.25)	
Median (Q1, Q3)	5.2 (4.37, 6.2)	4.5 (3.9, 5.96)	5.5 (4.75, 6.3)	
Tail lenght				0.221
Mean (SD)	27.1 (5.51)	26.49 (5.87)	27.88 (4.98)	
Median (Q1, Q3)	26.05 (22.9, 30.97)	25.4 (22.3, 30.7)	27.8 (23.7, 31.73)	
% COMET				0.402
Mean (SD)	11.71 (3.91)	11.29 (3.81)	12.25 (4.02)	
Median (Q1, Q3)	10.16 (8.48, 15.36)	9.55 (8.41, 14.15)	11.82 (8.5, 16.08)	
% Apoptotic cells				0.013
Mean (SD)	1.09 (0.32)	0.99 (0.27)	1.22 (0.34)	
Median (Q1, Q3)	1.07 (0.87, 1.26)	0.97 (0.83, 1.14)	1.13 (0.99, 1.4)	
% of subjects with oxidative DNA damage ≥ 4				0.130
No	54 (67.5%)	34 (75.6%)	20 (57.1%)	
Yes	26 (32.5%)	11 (24.4%)	15 (42.9%)	

**Table 4:** Multivariable regression analyses: adjustment for group status, gender, age, smoking status, and BMI. Notes: Odds Ratio (OR), Risk Ratio (RR).

	%DNA Tail Buff			%DNA Tail Enz			Oxidative DNA Damage			Tail Moment		
Predictors	OR	CI	*p*	OR	CI	*p*	OR	CI	*p*	RR	CI	*p*
FF vs. controls	1.27	1.12–1.45	<0.001	1.29	1.09–1.53	0.003	1.23	1.10–1.39	<0.001	1.34	1.15–1.55	<0.001
M vs. F	0.94	0.82–1.08	0.387	0.87	0.73–1.04	0.116	0.92	0.82–1.03	0.134	0.86	0.73–1.00	0.041
AGE	1.00	1.00–1.01	0.293	1.00	0.99–1.01	0.956	1.00	1.00–1.01	0.615	1.00	0.99–1.01	0.534
Smoking habits: Ex vs. No	1.11	0.98–1.26	0.103	0.95	0.80–1.12	0.508	1.01	0.91–1.12	0.821	1.11	0.96–1.29	0.123
Smoking habits: Yes vs. No	1.03	0.92–1.17	0.577	0.93	0.80–1.09	0.369	0.99	0.90–1.09	0.823	1.00	0.87–1.15	0.855
BMI	0.99	0.98–1.01	0.500	1.00	0.98–1.02	0.966	1.00	0.98–1.01	0.706	1.00	0.98–1.02	0.492
Fruit/vegetables: Yes vs. No	0.85	0.72–1.00	0.048	0.77	0.63–0.95	0.015	0.84	0.73–0.97	0.013	0.80	0.65–0.96	0.021
Grilled/smoked food: Yes vs. No	1.02	0.92–1.14	0.699	1.03	0.89–1.18	0.692	1.02	0.93–1.11	0.699	1.01	0.89–1.15	0.861
	Tail Lenght			% COMETS			% Apoptotic Cells					
Predictors	RR	CI	*p*	OR	CI	*p*	OR	CI	*p*			
FF vs. controls	1.20	1.06–1.35	0.004	1.16	0.95–1.43	0.154	1.46	1.24–1.71	<0.001			
M vs. F	0.96	0.84–1.09	0.505	0.95	0.76–1.18	0.628	0.88	0.74–1.04	0.120			
AGE	1.00	1.00–1.01	0.600	1.01	1.00–1.01	0.206	1.00	1.00–1.01	0.294			
Smoking habits: Ex vs. No	1.02	0.91–1.16	0.691	0.97	0.79–1.20	0.809	1.12	0.96–1.31	0.141			
Smoking habits: Yes vs. No	1.01	0.90–1.13	0.912	1.18	0.98–1.42	0.089	1.01	0.88–1.18	0.850			
BMI	0.99	0.97–1.01	0.179	1.00	0.97–1.03	0.887	0.99	0.97–1.01	0.382			
Fruit/vegetables: Yes vs. No	0.86	0.73–1.01	0.061	0.80	0.62–1.03	0.085	0.92	0.75–1.12	0.393			
Grilled/smoked food: Yes vs. No	0.96	0.86–1.07	0.433	0.93	0.78–1.11	0.436	0.87	0.76–1.00	0.050			

**Table 5:** DNA damage assessed by Comet assay. Differences between controls (CTRL), FFNE to fires in the previous 2 months, and FFE to fires in the last 2 months (FFE). SD = Standard Deviation; Q1 = First Quartile; Q3 = Third Quartile.

	Total (N = 80)	CTRL (N = 45)	FFNE (N = 9)	FFE (N = 26)	*p* Value
%DNA tail Buff					0.072
Mean (SD)	18.526 (3.546)	17.547 (3.352)	19.331 (2.778)	19.943 (3.666)	
Median (Q1, Q3)	18.200 (16.175, 20.350)	16.800 (15.300, 20.000)	19.600 (18.000, 20.900)	19.300 (18.000, 21.475)	
%DNA tail Enz					0.072
Mean (SD)	21.711 (5.140)	20.538 (4.814)	23.977 (3.588)	22.958 (5.713)	
Median (Q1, Q3)	21.400 (17.975, 25.000)	19.300 (17.500, 23.000)	24.900 (20.900, 25.200)	22.000 (20.125, 27.275)	
Oxidative DNA damage					0.571
Mean (SD)	3.185 (4.054)	2.991 (3.411)	4.646 (5.083)	3.015 (4.725)	
Median (Q1, Q3)	2.800 (0.700, 4.425)	2.700 (1.100, 4.000)	3.990 (1.700, 6.900)	2.900 (0.425, 4.990)	
Tail Moment					0.140
Mean (SD)	5.351 (1.398)	5.097 (1.468)	5.547 (1.269)	5.722 (1.262)	
Median (Q1, Q3)	5.200 (4.375, 6.200)	4.500 (3.900, 5.960)	5.800 (4.500, 6.300)	5.450 (4.825, 6.275)	
Tail length					0.496
Mean (SD)	27.098 (5.513)	26.486 (5.875)	27.967 (6.178)	27.856 (4.641)	
Median (Q1, Q3)	26.050 (22.900, 30.975)	25.400 (22.300, 30.700)	25.300 (23.400, 32.000)	28.150 (24.775, 31.325)	
% COMETS					0.558
Mean (SD)	11.708 (3.911)	11.288 (3.813)	11.451 (3.715)	12.525 (4.159)	
Median (Q1, Q3)	10.160 (8.477, 15.363)	9.550 (8.410, 14.150)	11.530 (8.160, 14.110)	12.380 (9.000, 16.457)	
% Apoptotic cells					0.045
Mean (SD)	1.091 (0.323)	0.987 (0.269)	1.140 (0.330)	1.253 (0.344)	
Median (Q1, Q3)	1.070 (0.870, 1.263)	0.970 (0.830, 1.140)	1.120 (0.960, 1.290)	1.140 (1.073, 1.453)	
% of subjects with oxidative DNA damage ≥ 4					0.347
No	54 (67.5%)	34 (75.6%)	5 (55.6%)	15 (57.7%)	
Yes	26 (32.5%)	11 (24.4%)	4 (44.4%)	11 (42.3%)	

**Table 6:** DNA damage assessed by BMcyt assay. Differences between non-exposed and exposed workers. SD = Standard Deviation; Q1 = First Quartile; Q3 = Third Quartile.

	Total (N = 80)	CTRL (N = 45)	VVF (N = 35)	*p* Value
‰MN				0.622
Mean (SD)	0.25 (0.53)	0.23 (0.5)	0.28 (0.57)	
Median (Q1, Q3)	0 (0, 0.45)	0 (0, 0)	0 (0, 0.47)	
N-Miss	3	3	0	
‰ Cells without nucleus				0.579
Mean (SD)	77.24 (35.34)	79.67 (34.94)	74.31 (36.09)	
Median (Q1, Q3)	67.1 (48.77, 96.03)	74.38 (54.15, 94.85)	60.63 (47.49, 95.1)	
N-Miss	3	3	0	
‰ Binucleated cells				0.723
Mean (SD)	2.7 (1.4)	2.56 (1.14)	2.86 (1.65)	
Median (Q1, Q3)	2.36 (1.81, 3.3)	2.36 (1.81, 3.38)	2.37 (1.84, 3.3)	
N-Miss	3	3	0	
‰Nuclear buds				0.117
Mean (SD)	0.7 (0.92)	0.51 (0.82)	0.93 (0.99)	
Median (Q1, Q3)	0.45 (0, 0.95)	0.22 (0, 0.93)	0.93 (0, 1.41)	
N-Miss	3	3	0	
‰Broken eggs				0.723
Mean (SD)	0.46 (0.75)	0.41 (0.64)	0.53 (0.87)	
Median (Q1, Q3)	0 (0, 0.9)	0 (0, 0.8)	0 (0, 0.7)	
N-Miss	3	3	0	
‰Condensed chromatin				0.047
Mean (SD)	0.24 (0.65)	0.06 (0.19)	0.46 (0.9)	
Median (Q1, Q3)	0 (0, 0)	0 (0, 0)	0 (0, 0.47)	
N-Miss	3	3	0	
‰Cell with more MN				0.852
Mean (SD)	0.05 (0.2)	0.037 (0.156)	0.06 (0.25)	
Median (Q1, Q3)	0 (0, 0)	0 (0, 0)	0 (0, 0)	
N-Miss	3	3	0	
‰Tot anomalies				0.047
Mean (SD)	4.42 (2.27)	3.82 (2.14)	5.15 (2.23)	
Median (Q1, Q3)	4.07 (2.82, 5.6)	3.61 (2.25, 4.73)	5.39 (3.5, 6.1)	
N-Miss	3	3	0	
‰Nucl bud + broken eggs				0.117
Mean (SD)	1.17 (1.25)	0.92 (1.16)	1.46 (1.31)	
Median (Q1, Q3)	0.9 (0, 1.87)	0.47 (0, 1.66)	1.3 (0.42, 2.35)	
N-Miss	3	3	0	
‰MN + ‰Nubud + ‰Brok eggs				0.117
Mean (SD)	1.42 (1.33)	1.15 (1.24)	1.74 (1.37)	
Median (Q1, Q3)	1.26 (0, 2.29)	0.89 (0, 1.86)	1.41 (0.68, 2.53)	
N-Miss	3	3	0	
MN positive subjects (cut off 1.5)				0.896
N-Miss	3	3	0	
NEG	75 (97.4%)	41 (97.6%)	34 (97.1%)	
POS	2 (2.6%)	1 (2.4%)	1 (2.9%)	

## Data Availability

The original contributions presented in this study are included in the article/Supplementary Materials. Further inquiries can be directed to the corresponding author.

## Supplementary Materials

The following supporting information can be downloaded at: https://www.mdpi.com/article/10.3390/jox16030078/s1. Figure S1: Functional enrichment analysis of predicted and validated miR-125b targets. Functional enrichment analysis was performed using the Enrichr platform. The bar chart displays the top enriched terms across three databases: Gene Ontology (GO) Biological Process (pink), MGI Mammalian Phenotype (orange), and KEGG Pathways (gray). Terms are ranked by their statistical significance, expressed as the −log10 (*p*-value)calculated via Fisher’s exact test. Targets are primarily involved in the regulation of transcription by RNA polymerase II (GO:0006357) and protein phosphorylation (GO:0006468). The analysis identifies key signaling axes, including MAPK and TGF-beta pathways, alongside neurotrophin signaling. Phenotypic enrichment (MGI) specifically highlights abnormal CNS synaptic transmission (MP:0002206) and embryonic lethality during organogenesis; Figure S2: Functional enrichment analysis of predicted and validated miR-29a targets. Functional enrichment analysis was performed using the Enrichr platform. The bar chart displays the top enriched terms across three databases: Gene Ontology (GO) Biological Process (pink), MGI Mammalian Phenotype (orange), and KEGG Pathways (gray). Terms are ranked by their statistical significance, expressed as the −log10 (*p*-value)calculated via Fisher’s exact test. Enrichment results demonstrate a high concentration of targets associated with structural integrity, specifically collagen fibril organization (GO:0030199) and extracellular matrix organization (GO:0030198). miR-29a deregulation is a typical signal of tissue remodeling or fibrosis processes. Significant KEGG pathways include Focal adhesion, AGE-RAGE signaling, and Pathways in cancer. Associated mammalian phenotypes (MGI) point toward abnormal blood vessel morphology (MP:0001614), absent kidney (MP:0000520), and decreased body size; Figure S3: Functional enrichment analysis of predicted and validated miR-181a targets. Functional enrichment analysis was performed using the Enrichr platform. The bar chart displays the top enriched terms across three databases: Gene Ontology (GO) Biological Process (pink), MGI Mammalian Phenotype (orange), and KEGG Pathways (gray). Terms are ranked by their statistical significance, expressed as the −log10 (*p*-value) calculated via Fisher’s exact test. Targets are primarily involved in the regulation of transcription by RNA polymerase II (GO:0006357) and in the positive regulation of cell differentiation (GO:0045597). KEGG analysis reveals enrichment in ubiquitin-mediated proteolysis, regulation of actin cytoskeleton, and signaling pathways regulating pluripotency of stem cells. MGI phenotypic data show a strong correlation with neonatal and postnatal lethality (MP:0011087, MP:0011085) and embryonic growth retardation (MP:0003984); Figure S4: Functional enrichment analysis of predicted and validated miR-142 targets. Functional enrichment analysis was performed using the Enrichr platform. The bar chart displays the top enriched terms across three databases: Gene Ontology (GO) Biological Process (pink), MGI Mammalian Phenotype (orange), and KEGG Pathways (gray). Terms are ranked by their statistical significance, expressed as the −log10 (*p*-value)calculated via Fisher’s exact test. Targets of miR-142 are significantly enriched in metabolic homeostasis pathways, including insulin resistance, insulin signaling, and FoxO signaling. Biological processes are centered on the negative regulation of transcription (GO:0045892) and protein phosphorylation (GO:0006468). Mammalian phenotype enrichment (MGI) identifies critical links to premature death (MP:0002083), perinatal lethality (MP:0011090), and reductions in body weight and size; Figure S5: Functional enrichment analysis of predicted and validated miR-10b targets. Functional enrichment analysis was performed using the Enrichr platform. The bar chart displays the top enriched terms across three databases: Gene Ontology (GO) Biological Process (pink), MGI Mammalian Phenotype (orange), and KEGG Pathways (gray). Terms are ranked by their statistical significance, expressed as the −log10 (*p*-value)calculated via Fisher’s exact test. Functional enrichment highlights a primary role in the regulation of transcription, DNA-templated (GO:0006355) and RNA polymerase II-dependent transcription (GO:0006357). Significant signaling pathways identified via KEGG include the Hippo signaling pathway, TGF-beta signaling pathway, two master regulators of organ size, cell proliferation, and epithelial-mesenchymal transition (EMT), and the FoxO signaling pathway. Phenotypic data from MGI suggest that disruption of miR-10b targets is associated with preweaning lethality (MP:0011110), decreased embryo size (MP:0001698), and reduced long-term potentiation (MP:0001473), pointing toward essential functions in both systemic development and neuronal homeostasis; Figure S6: Functional enrichment analysis of predicted and validated miR-16 targets. Functional enrichment analysis was performed using the Enrichr platform. The bar chart displays the top enriched terms across three databases: Gene Ontology (GO) Biological Process (pink), MGI Mammalian Phenotype (orange), and KEGG Pathways (gray). Terms are ranked by their statistical significance, expressed as the −log10 (*p*-value) calculated via Fisher’s exact test. Biological processes are characterized by the regulation of transcription (GO:0006357, GO:0006355) and a highly specific enrichment in 3′-UTR-mediated mRNA stabilization (GO:0070935) and Golgi organization (GO:0007030). KEGG analysis identifies ErbB signaling, Focal adhesion, and Pathways in cancer as primary regulatory targets. This profile underscores miR-16′s established role as a master regulator of the cell cycle and proliferation. Phenotypic enrichment (MGI) underscores miR-16′s critical role in neuromuscular integrity and development, highlighting abnormal motor neuron innervation pattern (MP:0000940), impaired coordination (MP:0001405), and embryonic lethality (MP:0011098, MP:0011092); Figure S7: Functional enrichment analysis of predicted and validated miR-15a targets. Functional enrichment analysis was performed using the Enrichr platform. The bar chart displays the top enriched terms across three databases: Gene Ontology (GO) Biological Process (pink), MGI Mammalian Phenotype (orange), and KEGG Pathways (gray). Terms are ranked by their statistical significance, expressed as the −log10 (*p*-value) calculated via Fisher’s exact test. Biological processes (GO) are dominated by metabolic detoxification pathways, including flavonoid glucuronidation (GO:0052696), xenobiotic glucuronidation (GO:0052697), and cellular glucuronidation (GO:0052695). miR 15-a targets are heavily enriched in pathways governing cell cycle progression and proliferative signaling, such as ErbB and T cell receptor signaling. Mammalian phenotype enrichment (MGI) reveals critical roles in structural and endocrine homeostasis, specifically abnormal myocardium layer morphology (MP:0005329), abnormal osteoclast differentiation (MP:0008396), and increased circulating prolactin level (MP:0005124), alongside decreased body length (MP:0001258); Figure S8: Comparative functional enrichment heatmap across the miRNA study set. This heatmap, generated through the Metascape integrative analysis pipeline, provides a comparative visualization of the functional themes associated with the target genes of miR-181a, miR-125a, miR-15a, miR-142, miR-10b, miR-16, and miR-29a. Each cell’s color intensity reflects the statistical significance of enrichment, quantified as the −log10 (*p*-value). The scale ranges from 0 (light yellow, non-significant) to a maximum of 20 (dark orange, highly significant), with gray cells indicating a lack of enrichment for that specific term. The y-axis features hierarchical clustering of enriched terms based on semantic similarity, identifying major functional blocks shared across the miRNA panel. A dominant cluster is observed for cell population proliferation (GO:0008283) and regulation of growth (GO:0040008), where nearly all miRNAs exhibit high-significance scores (deep orange), confirming their collective role in coordinating cell cycle progression and tissue expansion. Furthermore, the consistent enrichment in Pathways in cancer (hsa05200) and the MAPK cascade (GO:0000165) highlights a conserved regulatory axis focused on signal transduction and proliferative stimuli. Statistical significance was determined using Fisher’s exact test, followed by a Benjamini-Hochberg adjustment for multiple testing. The analysis integrated multiple ontological sources, including GO Biological Process, KEGG Pathways, and WikiPathways, ensuring a robust multi-dimensional characterization of the miRNA-regulated interactome; Figure S9: Functional overlap and miRNA contribution within the enrichment network. This network, generated via the Metascape Gene List Analysis Pipeline, visualizes the connectivity between enriched biological terms for the studied miRNA panel. Each node represents an enriched term, with the node size proportional to the number of target genes. Nodes are displayed as pie charts, where colors represent the relative contribution of each miRNA to that specific term: miR-10b (red), miR-125a (blue), miR-142 (green), miR-15a (purple), miR-16 (orange), miR-29a (yellow), and miR-181a (brown). The high density of multi-colored nodes demonstrates a significant functional synergy, suggesting that these miRNAs act as a coordinated regulatory unit rather than influencing isolated pathways. Statistical significance was determined using Fisher’s exact test; Figure S10: Modular architecture of the enriched miRNA-target interactome. This visualization, generated via the Metascape Gene List Analysis Pipeline, represents the same enrichment network, with nodes colored according to functional cluster identity to highlight semantic similarities between pathways. Distinct color-coded modules identify key regulatory hubs, including enzyme-linked receptor signaling, neuron projection development, and the MAPK cascade. Edges connect nodes with a similarity score (Kappa index) > 0.3, revealing how diverse processes such as tubule morphogenesis and cell population proliferation are molecularly linked through shared target genes. This layout underscores the master regulatory role of the miRNA panel in orchestrating complex biological transitions and systemic growth. Statistical significance was determined using Fisher’s exact test. The results of this study are based on the data provided in the dataset included in the supplementary materials.

## Abbreviations

The following abbreviations are used in this manuscript:

FF	Firefighters
PAHs	Polycyclic aromatic hydrocarbons
FA	Formaldehyde
IARC	International Agency for Research on Cancer
MN	Micronucleus
miRNA	microRNA
Fpg	formamidopyrimidine DNA glycosylase
BMCyt	Buccal micronucleus Cytome
NB	Nuclear buds
BE	Broken egg
FFNE	Firefighters not exposed
FFE	Firefighters exposed
BMI	Body mass index
