# Supplementary material for: Genotoxic Damage and microRNA Dysregulation in Firefighters: An Integrated Biomonitoring Case Study
Source: J Xenobiot. 2026 May 5;16(3):78. doi: 10.3390/jox16030078 (PMC13214885; doi:10.3390/jox16030078)
Supplement: Supplementary file 1 [file jox-16-00078-s001.zip › jox-4224501-Supplementary material.pdf]

Journal of *Xenobiotics*

MDPI

Supplementary Materials: Genotoxic Damage and microRNA Dysregulation in Firefighters: An Integrated Biomonitoring Case Study

Claudia Cipollone, Riccardo Mastrantonio, Paola Mozzoni, Giada Mastrangeli, Massimo Corradi, Stefano Renzetti, Veronica Saponara, Maria Nicastro, Delia Cavallo, Raffaele Maiello, Marco Gentile, Diana Poli, Mario Muselli, Alessia Romantini, Giorgia Di Gennaro, Gloria Cenci, Carmela Protano, Matteo Vitali, Giuseppe De Palma, Cinzia Lucia Ursini, and Leila Fabiani

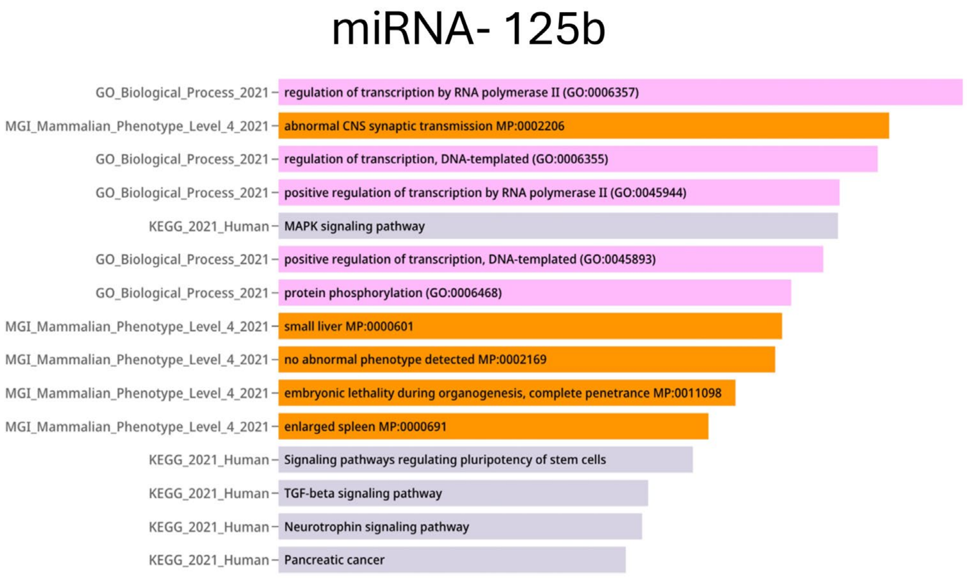

Figure S1:Functional enrichment analysis of predicted and validated miR-125b targets. Functional enrichment analysis was performed using the Enrichr platform. The bar chart displays the top enriched terms across three databases: Gene Ontology (GO) Biological Process (pink), MGI Mammalian Phenotype (orange) and KEGG Pathways (gray). Terms are ranked by their statistical significance, expressed as the –log10 (p value)calculated via Fisher's exact test. Targets are primarily involved in the regulation of transcription by RNA polymerase II (GO:0006357) and protein phosphorylation (GO:0006468). The analysis identifies key signaling axes including MAPK and TGF-beta pathways, alongside neurotrophin signaling. Phenotypic enrichment (MGI) specifically highlights abnormal CNS synaptic transmission (MP:0002206) and embryonic lethality during organogenesis

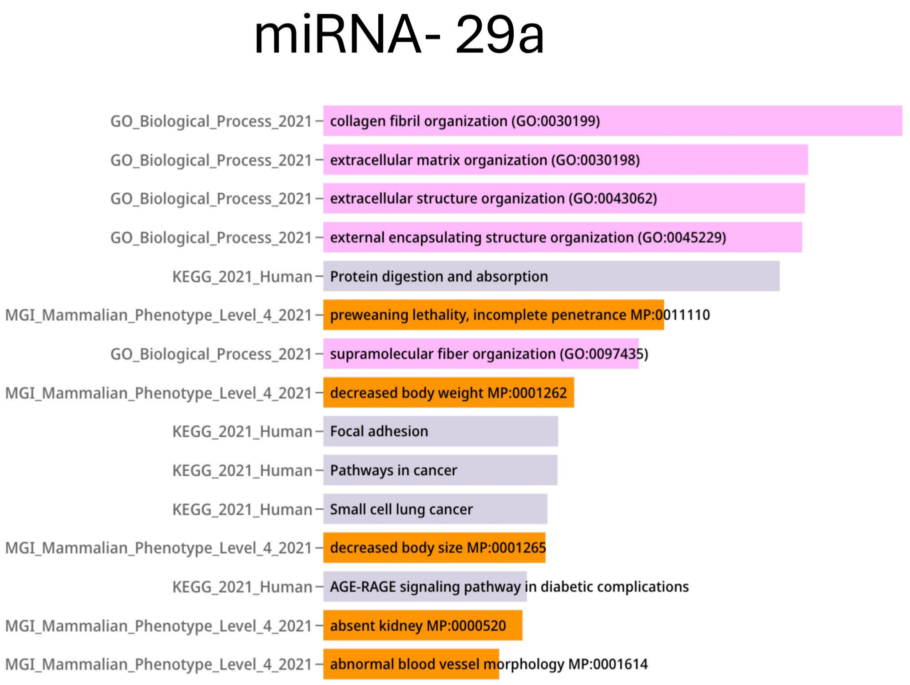

Figure S2: Functional enrichment analysis of predicted and validated miR-29a targets . Functional enrichment analysis was performed using the Enrichr platform. The bar chart displays the top enriched terms across three databases: Gene Ontology (GO) Biological Process (pink), MGI Mammalian Phenotype (orange) and KEGG Pathways (gray). Terms are ranked by their statistical significance, expressed as the –log10 (p value)calculated via Fisher's exact test. Enrichment results demonstrate a high concentration of targets associated with structural integrity, specifically collagen fibril organization (GO:0030199) and extracellular matrix organization (GO:0030198). miR-29a deregulation is a typical signal of tissue remodeling or fibrosis processes. Significant KEGG pathways include Focal adhesion, AGE-RAGE signaling, and Pathways in cancer. Associated mammalian phenotypes (MGI) point toward abnormal blood vessel morphology (MP:0001614), absent kidney (MP:0000520), and decreased body size.

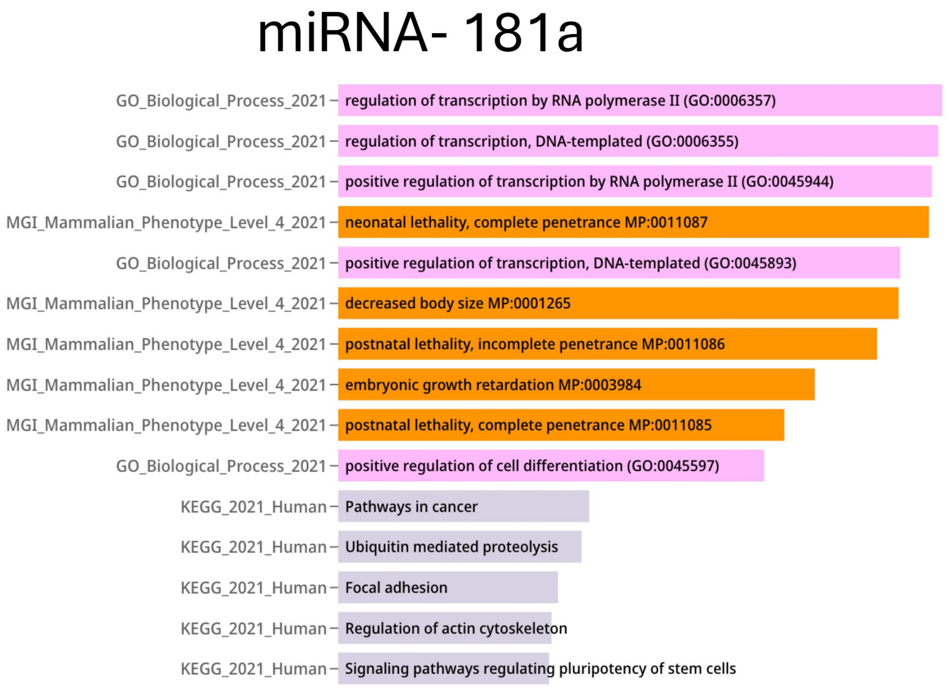

Figure S3:Functional enrichment analysis of predicted and validated miR-181a targets Functional enrichment analysis was performed using the Enrichr platform. The bar chart displays the top enriched terms across three databases: Gene Ontology (GO) Biological Process (pink), MGI Mammalian Phenotype (orange) and KEGG Pathways (gray). Terms are ranked by their statistical significance, expressed as the –log10 (p value) calculated via Fisher's exact test. Targets are primarily involved in the regulation of transcription by RNA polymerase II (GO:0006357) and in the positive regulation of cell differentiation (GO:0045597). KEGG analysis reveals enrichment in ubiquitin mediated proteolysis, regulation of actin cytoskeleton, and signaling pathways regulating pluripotency of stem cells. MGI phenotypic data show a strong correlation with neonatal and postnatal lethality (MP:0011087, MP:0011085) and embryonic growth retardation (MP:0003984).

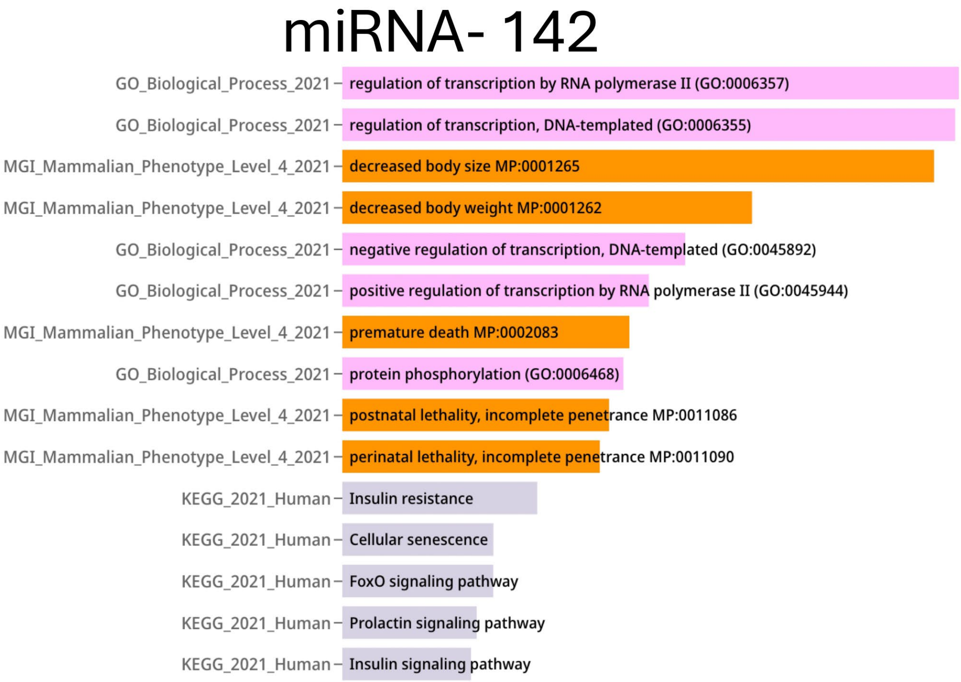

Figure S4: Functional enrichment analysis of predicted and validated miR-142 targets . Functional enrichment analysis was performed using the Enrichr platform. The bar chart displays the top enriched terms across three databases: Gene Ontology (GO) Biological Process (pink), MGI Mammalian Phenotype (orange) and KEGG Pathways (gray). Terms are ranked by their statistical significance, expressed as the –log10 (p value)calculated via Fisher's exact test. Targets of miR-142 are significantly enriched in metabolic homeostasis pathways, including insulin resistance, insulin signaling and FoxO signaling. Biological processes are centered on the negative regulation of transcription (GO:0045892) and protein phosphorylation (GO:0006468). Mammalian phenotype enrichment (MGI) identifies critical links to premature death (MP:0002083), perinatal lethality (MP:0011090) and reductions in body weight and size.

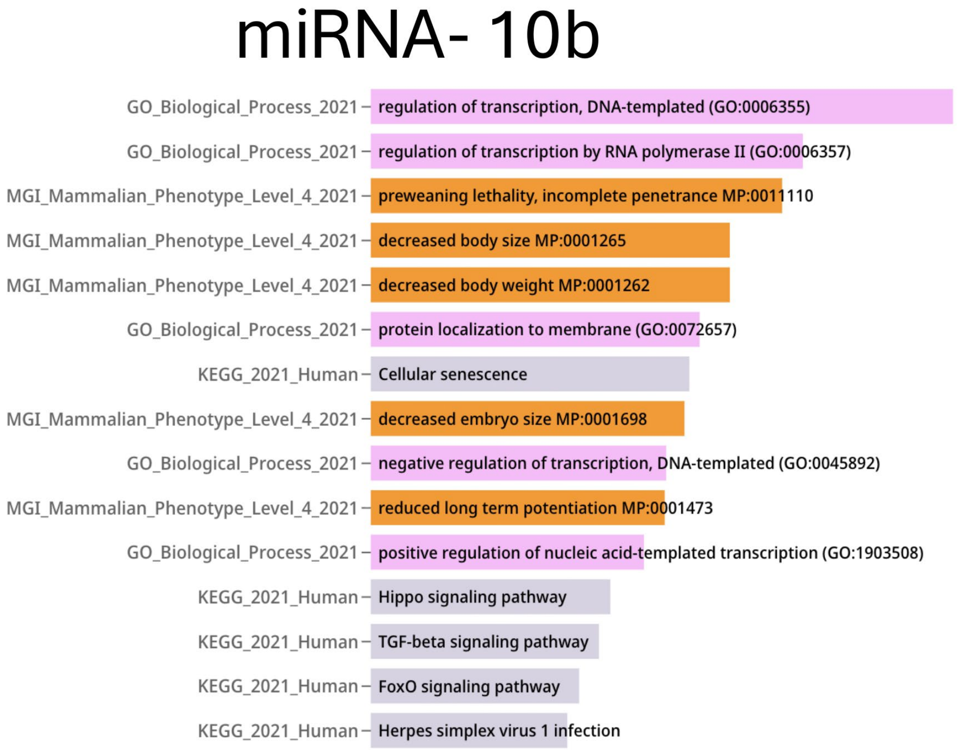

Figure S5: Functional enrichment analysis of predicted and validated miR-10b targets. Functional enrichment analysis was performed using the Enrichr platform. The bar chart displays the top enriched terms across three databases: Gene Ontology (GO) Biological Process (pink), MGI Mammalian Phenotype (orange) and KEGG Pathways (gray). Terms are ranked by their statistical significance, expressed as the –log10 (p value)calculated via Fisher's exact test. Functional enrichment highlights a primary role in the regulation of transcription, DNA-templated (GO:0006355) and RNA polymerase II-dependent transcription (GO:0006357). Significant signaling pathways identified via KEGG include the Hippo signaling pathway, TGF-beta signaling pathway, two master regulators of organ size, cell proliferation, and epithelial-mesenchymal transition (EMT), and FoxO signaling pathway. Phenotypic data from MGI suggests that disruption of miR-10b targets is associated with preweaning lethality (MP:0011110), decreased embryo size (MP:0001698) and reduced long-term potentiation (MP:0001473), pointing toward essential func-tions in both systemic development and neuronal homeostasis.

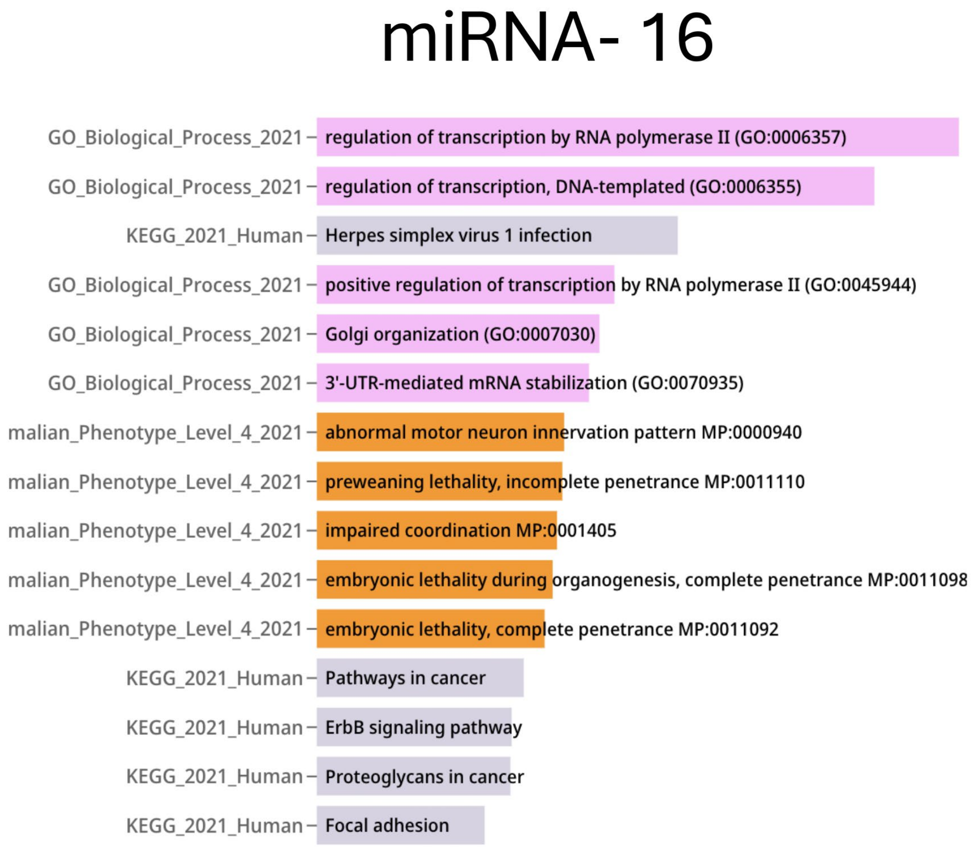

Figure S6: Functional enrichment analysis of predicted and validated miR-16 targets. Functional enrichment analysis was performed using the Enrichr platform. The bar chart displays the top enriched terms across three databases: Gene Ontology (GO) Biological Process (pink), MGI Mammalian Phenotype (orange) and KEGG Pathways (gray). Terms are ranked by their statistical significance, expressed as the –log10 (p value) calculated via Fisher's exact test. Biological processes are characterized by the regulation of transcription (GO:0006357, GO:0006355) and a highly specific enrichment in 3'-UTR-mediated mRNA stabilization (GO:0070935) and Golgi organization (GO:0007030). KEGG analysis identifies ErbB signaling, Focal adhesion and Pathways in cancer as primary regulatory targets. This profile underscores miR-16's established role as a master regu-lator of the cell cycle and proliferation. Phenotypic enrichment (MGI) underscores miR-16's critical role in neuromuscular integrity and development, highlighting abnormal motor neuron innervation pattern (MP:0000940), impaired coordination (MP:0001405) and embryonic lethality (MP:0011098, MP:0011092).

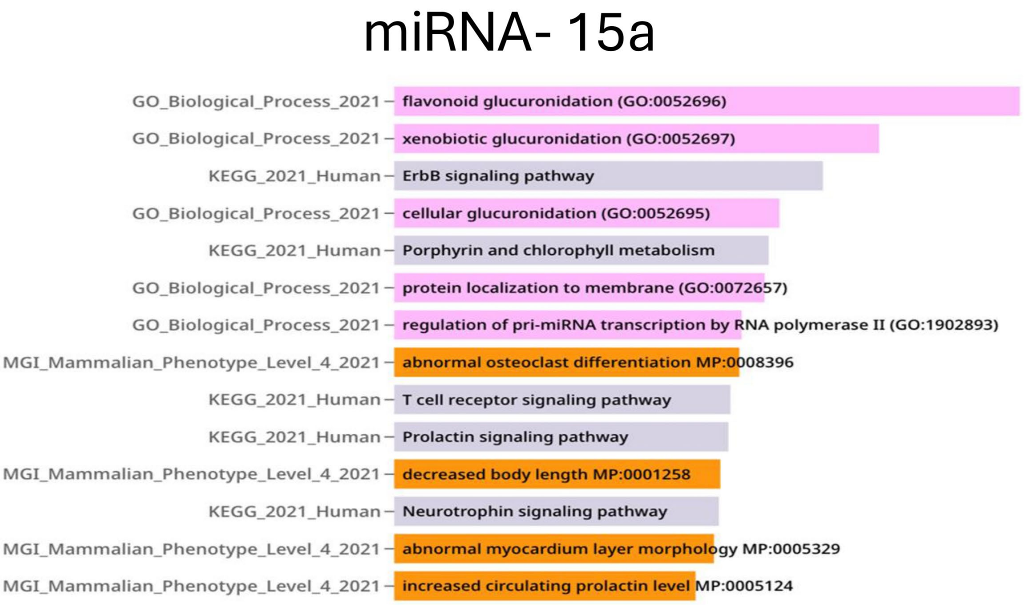

Figure S7: Functional enrichment analysis of predicted and validated miR-15a targets. Functional enrichment analysis was performed using the Enrichr platform. The bar chart displays the top enriched terms across three databases: Gene Ontology (GO) Biological Process (pink), MGI Mammalian Phenotype (orange) and KEGG Pathways (gray). Terms are ranked by their statistical significance, expressed as the –log10 (p value) calculated via Fisher's exact test. Biological processes (GO) are dominated by metabolic detoxification pathways, including flavonoid glucuronidation (GO:0052696), xenobiotic glucuronidation (GO:0052697), and cellular glucuronidation (GO:0052695). miR 15-a targets are heavily enriched in pathways governing cell cycle progression and proliferative signaling, such as ErbB and T cell receptor signaling. Mammalian phenotype enrichment (MGI) reveals critical roles in structural and endocrine homeostasis, specifically ab-normal myocardium layer morphology (MP:0005329), abnormal osteoclast differentiation (MP:0008396), and increased circulating prolactin level (MP:0005124), alongside decreased body length (MP:0001258).

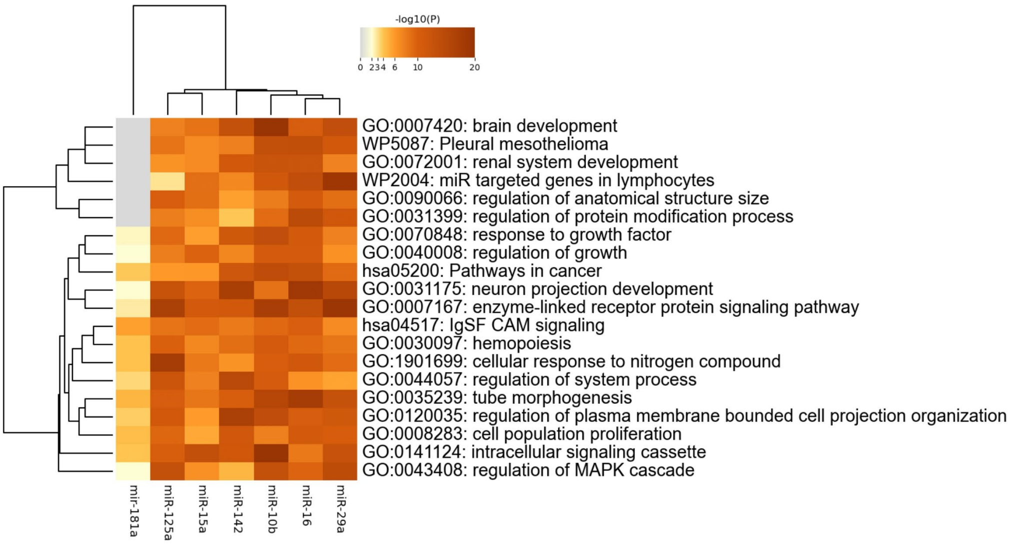

Figure S8: Comparative functional enrichment heatmap across the miRNA study set. This heatmap, generated through the Metascape integrative analysis pipeline, provides a comparative visualization of the functional themes associated with the target genes of miR-181a, miR-125a, miR-15a, miR-142, miR-10b, miR-16 and miR-29a. Each cell's color intensity reflects the statistical significance of enrichment, quantified as the –log10 (p value). The scale ranges from 0 (light yellow, non-significant) to a maximum of 20 (dark orange, highly significant), with gray cells indicating a lack of enrichment for that specific term. The y-axis features hierarchical clustering of enriched terms based on semantic similarity,

identifying major functional blocks shared across the miRNA panel. A dominant cluster is observed for cell population proliferation (GO:0008283) and regula-tion of growth (GO:0040008), where nearly all miRNAs exhibit high-significance scores (deep orange), confirming their collective role in coordinating cell cycle progression and tissue expan-sion. Furthermore, the consistent enrichment in Pathways in cancer (hsa05200) and the MAPK cascade (GO:0000165) highlights a conserved regulatory axis focused on signal transduction and proliferative stimuli. Statistical significance was determined using the Fisher's exact test, followed by a Benjamini-Hochberg adjustment for multiple testing. The analysis integrated multiple on-tological sources, including GO Biological Process, KEGG Pathways and WikiPathways, ensuring a robust multi-dimensional characterization of the miRNA-regulated interactome.

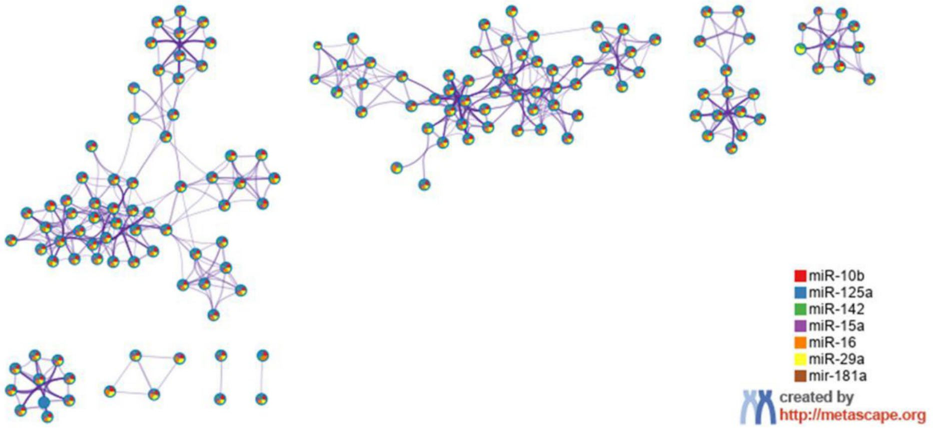

Figure S9: Functional overlap and miRNA contribution within the enrichment network. This network, generated via the Metascape Gene List Analysis Pipeline, visualizes the connectivity between enriched biological terms for the studied miRNA panel. Each node represents an enriched term, with the node size proportional to the number of target genes. Nodes are displayed as pie charts, where colors represent the relative contribution of each miRNA to that specific term: miR-10b (red), miR-125a (blue), miR-142 (green), miR-15a (purple), miR-16 (orange), miR-29a (yellow), and miR-181a (brown). The high density of multi-colored nodes demonstrates a signif-icant functional synergy, suggesting that these miRNAs act as a coordinated regulatory unit rather than influencing isolated pathways. Statistical significance was determined using Fisher's exact test.

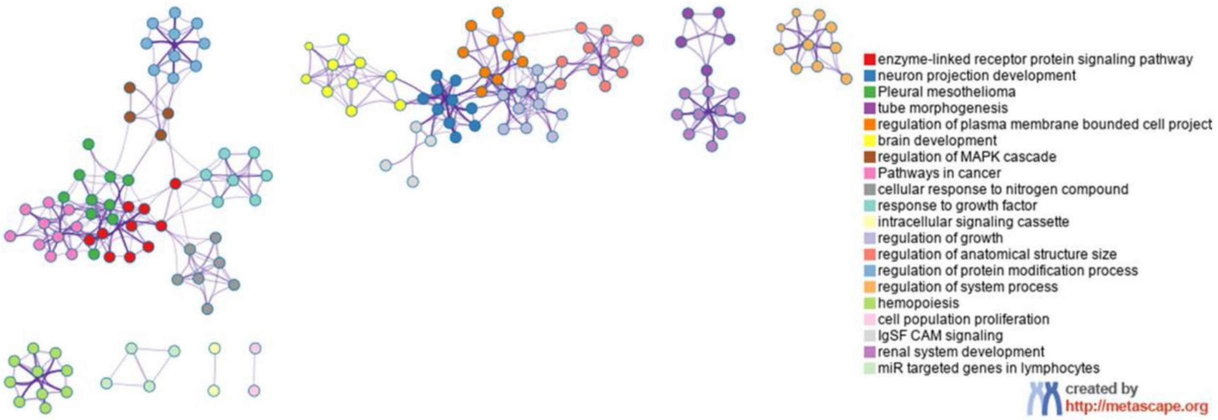

Figure S10: Modular architecture of the enriched miRNA-target interactome. This visualization, generated via the Metascape Gene List Analysis Pipeline, represents the same enrichment network, with nodes colored according to functional cluster identity to highlight semantic similarities between pathways. Distinct color-coded modules identify key regulatory hubs, including en-zyme-linked receptor signaling, neuron projection development, and the MAPK cascade. Edges connect nodes with a similarity score (Kappa index) > 0.3, revealing how diverse processes such as tubule morphogenesis and cell population proliferation are molecularly linked through shared target genes. This layout underscores the master regulatory role of the miRNA panel in orches-trating complex biological transitions and systemic growth. Statistical significance was deter-mined using Fisher’s exact test.

The results of this study are based on the data provided in the dataset included in the supple-mentary materials
